# NUMB negatively regulates the epithelial-mesenchymal transition of triple-negative breast cancer by antagonizing Notch signaling

**DOI:** 10.18632/oncotarget.11062

**Published:** 2016-08-05

**Authors:** Jianchao Zhang, Ximing Shao, Haiyan Sun, Ke Liu, Zhihao Ding, Juntao Chen, Lijing Fang, Wu Su, Yang Hong, Huashun Li, Hongchang Li

**Affiliations:** ^1^ Shenzhen Key Laboratory for Molecular Biology of Neural Development, Guangdong Key Laboratory of Nanomedicine, Institute of Biomedicine and Biotechnology, Shenzhen Institutes of Advanced Technology, Chinese Academy of Sciences, Shenzhen, Guangdong 518055, China; ^2^ SARITEX Center for Stem Cell Engineering Translational Medicine, Shanghai East Hospital, Tongji University School of Medicine and Advanced Institute of Translational Medicine, Shanghai 200123, China; ^3^ ATCG Corporation, BioBay, Suzhou Industrial Park, Suzhou, Jiangsu 215123, China; ^4^ Department of Cell Biology and Physiology, University of Pittsburgh School of Medicine, Pittsburgh, PA 15261, USA

**Keywords:** NUMB, EMT, TNBC, Notch, metastasis

## Abstract

Triple-negative breast cancer (TNBC), an aggressive subtype of breast cancer with higher rates of early relapse and metastasis, is frequently associated with aberrant activation of epithelial-mesenchymal transition (EMT). Nonetheless, how EMT is initiated and regulated during TNBC progression is not well understood. Here, we report that NUMB is a negative regulator of EMT in both human mammary epithelial cells and breast cancer cells. Reduced NUMB expression was significantly associated with elevated EMT in TNBC. Conversely, overexpression of NUMB strongly attenuated the EMT program and metastasis of TNBC cell lines. Interestingly, we showed that NUMB employs different molecular mechanisms to regulate EMT. In normal mammary epithelial cells and breast cancer cells expressing wild-type p53, NUMB suppressed EMT by stabilizing p53. However, in TNBC cells, loss of NUMB facilitated the EMT program by activating Notch signaling. Consistent with these findings, low NUMB expression and high Notch activity were significantly correlated with the TNBC subtype in patients. Collectively, these findings reveal novel molecular mechanisms of NUMB in the regulation of breast tumor EMT, especially in TNBC.

## INTRODUCTION

Triple-negative breast cancer (TNBC) represents a subgroup of breast cancers that is negative for estrogen receptors (ER), progesterone receptors (PR), and human epidermal growth factor receptor 2 (HER2) [1]. TNBC tends to exhibit aggressive and metastatic clinical behavior [1–3]. Epithelial-mesenchymal transition (EMT) is a developmental process in which epithelial cells lose polarity and acquire migratory and invasive properties to become mesenchymal cells [4]. During cancer progression, EMT confers stem cell-like characteristics on cancer cells to facilitate tumor metastasis [5–7]. In TNBC, the aberrant activation of the EMT program has been implicated in the initiation of metastasis and aggressive progression [8, 9]. Among various types of breast cancers, EMT markers are more frequently expressed in TNBC, further suggesting a strong association between the EMT program and TNBC progression [10, 11]. Therefore, inhibition of EMT may be a potential therapeutic strategy for TNBC patients.

NUMB, an evolutionarily conserved protein from fly to mammals, plays critical roles in cell fate determination, cell polarity maintenance, cell migration and endocytosis [12–16]. In addition to its role in the regulation of these physiological developmental processes, NUMB has been shown to be a tumor suppressor in cancer progression [17]. Loss of NUMB expression has been found in salivary gland carcinoma, malignant pleural mesothelioma, breast tumors, non-small cell lung cancer and esophageal squamous cell carcinoma [18–22]. Although the mechanisms underlying NUMB involvement in cancer are largely unknown, accumulating evidence suggests a critical role of NUMB in the EMT program. In neuron progenitor cells, NUMB interacts with CDH1 and CDH2 and controls the proper intracellular location of these crucial adhesion molecules [23]. Deletion of NUMB disrupts the adhesion between epithelial neuron progenitors and enables them to migrate [23]. NUMB is also involved in HGF-induced EMT via regulation of E-cadherin and the cell polarity molecules Par3, Par6, and aPkc [14]. Additionally, NUMB is linked to two well-studied EMT regulators, p53 and Notch [24–26]. Through interaction with MDM2, NUMB stabilizes p53 expression in breast cancer, liver cancer and renal fibrosis [20, 27, 28]. NUMB inhibits Notch signaling by promoting ubiquitination and degradation of the Notch1 intracellular domain (NICD) *in vitro* and in mouse cardiac development [29, 30]. Thus, loss of NUMB in tumors may contribute to EMT through a dual mechanism: activation of the p53 pathway and inhibition of the Notch pathway.

In the present study, we report that NUMB is a negative regulator of EMT in both human mammary epithelial cells and breast cancer cells. Interestingly, we found a specific correlation between reduced expression of NUMB and elevated EMT in TNBC cells. Overexpression of NUMB strikingly attenuated the EMT program and metastasis of TNBC cells. Moreover, we showed that NUMB employed different molecular mechanisms to negatively regulate EMT in different circumstances. In normal human mammary epithelial cells and breast cancer cells expressing wild-type p53, NUMB suppressed EMT by stabilizing p53. However, in TNBC cells, NUMB reduction facilitated the EMT process via activation of Notch signaling. Together, these findings reveal novel functions of NUMB in the regulation of breast tumor EMT, especially in the TNBC subtype.

## RESULTS

### NUMB knockdown promotes EMT in human mammary epithelial cells

**Figure 1:**
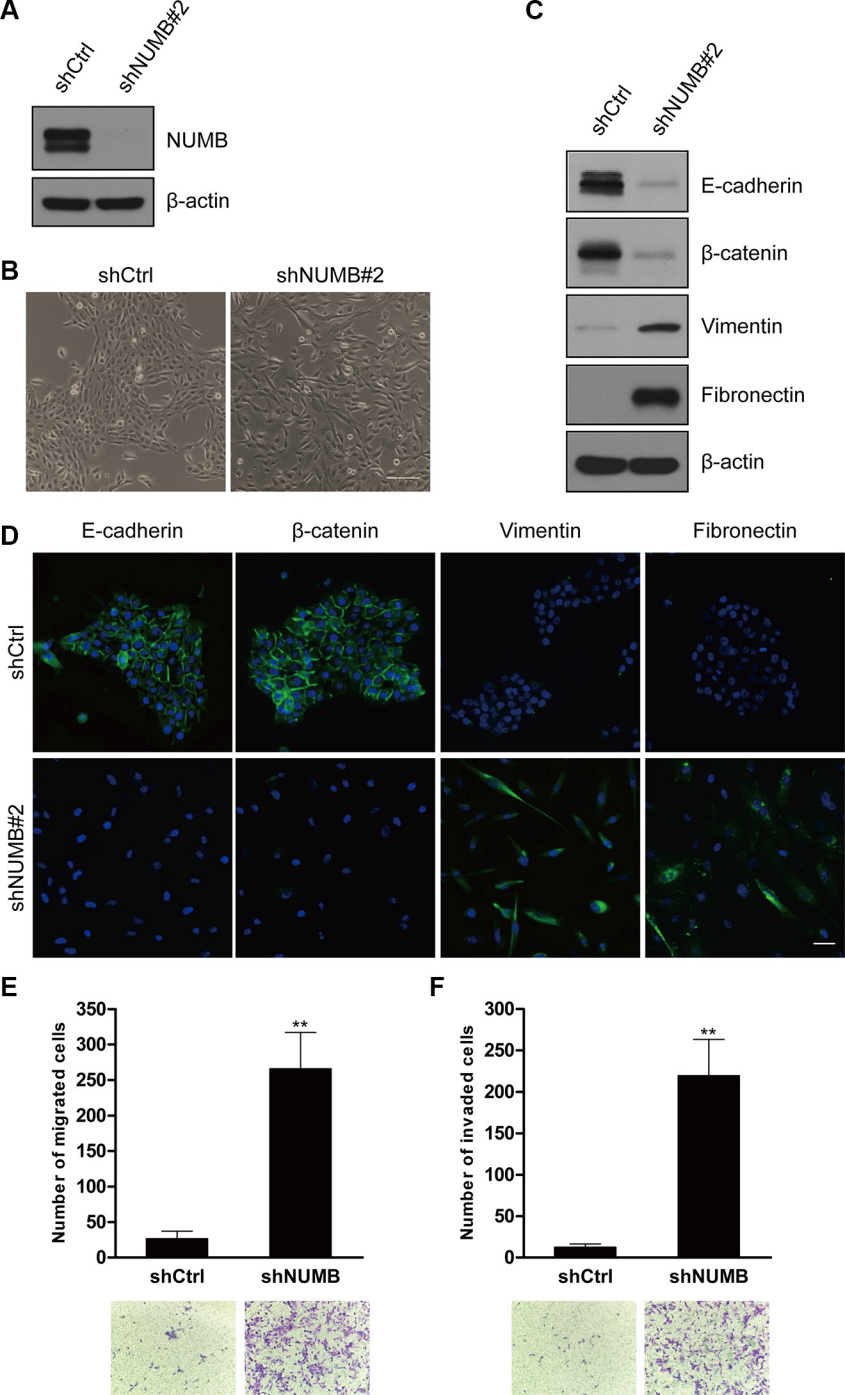
NUMB knockdown promotes EMT (**A**) Immunoblotting analysis of the NUMB gene knockdown after lentiviral infection in MCF10A cells. (**B**) Morphological changes in MCF10A cells after NUMB silencing. Scale bar = 100 μm. (**C**) Immunoblotting analysis of expression of the epithelial markers E-cadherin and β-catenin and the mesenchymal markers vimentin and fibronectin. (**D**) Immunofluorescence staining of the EMT markers at low cell density. Scale bar =100 μm. (**E**) Migration and (**F**) invasion assays of MCF10A cells after NUMB silencing. The mean was derived from cell counts in five fields, and each experiment was repeated three times (**p < 0.01, compared to the control). Representative images of migrated and invaded cells are shown.

To investigate the role of NUMB in EMT, we knocked down NUMB expression in the immortalized normal human mammary epithelial cell line MCF10A using lentiviral transduction, which was confirmed by immunoblotting (Figure 1A and Supplementary Figure S1A). After NUMB knockdown, MCF10A cells showed an elongated spindle-like morphology with a scattered distribution in culture, while cells expressing the control shRNA retained their cobblestone-like morphology with tight cell-cell adhesion (Figure 1B and Supplementary Figure S1B). Both epithelial and mesenchymal markers expression was confirmed by immunoblotting (Figure 1C and Supplementary Figure S1A), and immunofluorescence at low (Figure 1D) or high (Supplementary Figure S1C) cell density was assessed. Expression of the epithelial markers E-cadherin and β-catenin was significantly reduced in NUMB-knockdown cells, but expression of the mesenchymal markers fibronectin and vimentin was dramatically upregulated. These morphological and molecular changes suggested the transition of the NUMB-knockdown MCF10A cells from an epithelial to mesenchymal status. Typically, the EMT phenotype is usually accompanied by increased migration and invasion [31]. As shown in Figure 1E and 1F, knockdown of NUMB expression resulted in increased migratory and invasive behaviors in human mammary epithelial cells. Together, these results show that suppression of NUMB expression induces the EMT program.

### NUMB suppression induces stem cell-like phenotype

**Figure 2:**
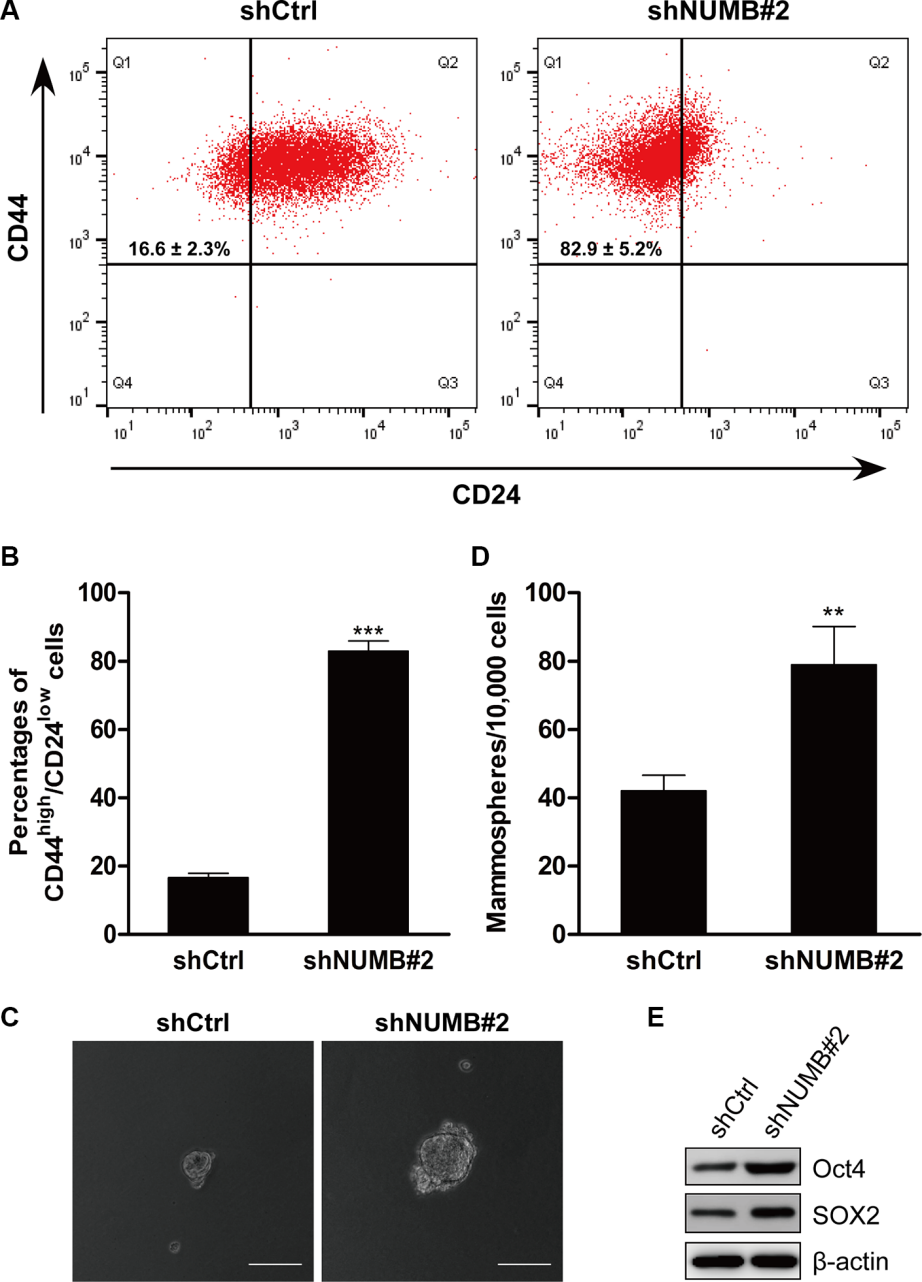
NUMB suppression induces stem cell-like phenotypes (**A**) FACS analysis of the cell-surface markers CD44 and CD24 in MCF10A cells after NUMB silencing. Percentages of the mean CD44^high^/CD24^low^ subpopulation ± SD based on triplicate experiments are indicated. (**B**) The percentage of the CD44^high^/CD24^low^ population in MCF10A cells after NUMB silencing is indicated in the bar graph. (**C**) Phase contrast images of mammosphere formation in MCF10A cells after NUMB silencing. Scale bar =100 μm. (**D**) Quantification of mammospheres formed in three independent experiments (error bar: mean ± SD) (***p < 0.001, **p < 0.01, compared to the control). (**E**) Immunoblotting analysis of expression of the stem cell markers Oct4 and SOX2.

Mammary epithelial cells that undergo EMT display stemness properties, such as an increased CD44^high^/CD24^low^ population and mammosphere formation [5]. To determine whether NUMB knockdown affects the stem cell phenotypes upon induction of EMT, we performed FACS to identify the CD44^high^/CD24^low^ populations. We found that the NUMB-knockdown MCF10A cells exhibited a significant increase in the CD44^high^/CD24^low^ stem cell population compared with their corresponding control cells (Figure 2A and 2B). Meanwhile, the NUMB-knockdown MCF10A cells displayed an increased size and number of mammospheres compared with the control cells (Figure 2C and 2D). Representative stem cell markers were analyzed by immunoblotting. As shown in Figure 2E, expression of the stem cell markers Oct4 and SOX2 was dramatically upregulated in NUMB-knockdown MCF10A cells. We thus conclude that the EMT induced by NUMB downregulation generates mesenchymal cells with stem cell-like phenotypes, a common feature of EMT regulators.

### NUMB suppresses EMT by stabilizing p53 in human mammary epithelial cells

**Figure 3:**
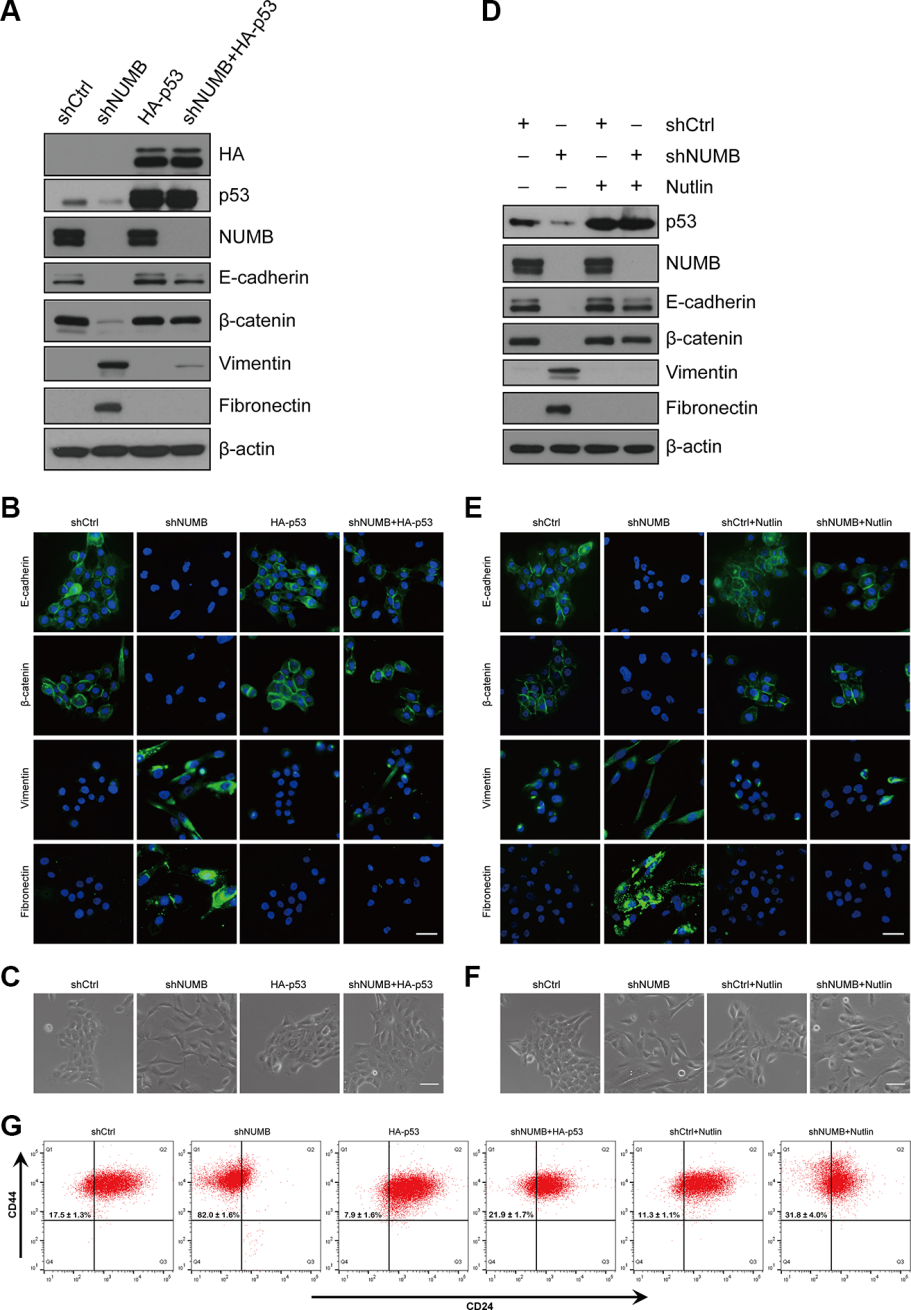
NUMB suppresses EMT by stabilizing p53 in MCF10A cells (**A**) Expression of NUMB, p53, HA and EMT markers was analyzed by immunoblotting in MCF10A cells expressing the indicated constructs. (**B**) Immunofluorescence staining of EMT markers in MCF10A cells expressing the indicated constructs. Scale bar = 100 μm. (**C**) Morphology of MCF10A cells expressing the indicated constructs. Scale bar = 50 μm. (**D**) Immunoblotting of p53, NUMB and EMT markers after treatment of Nutlin-3 (10 μM) in MCF10A cells with shCtrl and shNUMB. (**E**) Immunofluorescence staining of EMT markers after treatment with Nutlin-3 as in E. Scale bar =100 μm. (**F**) Morphology of MCF10A cells with shCtrl and shNUMB after Nutlin-3 treatment. Scale bar =50 μm. (**G**) FACS analysis of the cell-surface markers CD44 and CD24 in MCF10A cells as indicated. Percentages of the mean CD44^high^/CD24^low^ subpopulation ± SD based on triplicate experiments are indicated.

Previous reports have shown that NUMB is involved in tumor progression by controlling p53 stability [20, 32, 33]. As p53 is a well-documented EMT regulator [24, 34], we hypothesized that NUMB may regulate EMT via the p53 pathway. Indeed, NUMB knockdown dramatically reduced the p53 protein level in MCF10A cells (Supplementary Figure S2A). Further analysis showed that NUMB knockdown had no effect on p53 mRNA transcription, but it substantially increased the ubiquitination and degradation of p53 (Supplementary Figure S2B–S2D). These data are consistent with a previous report indicating that NUMB protects p53 from ubiquitin-mediated degradation [20]. We next determined whether reduced expression of p53 is responsible for NUMB knockdown-induced EMT. Thus, we exogenously expressed p53 in NUMB-knockdown MCF10A cells through lentiviral transduction. Immunoblotting and immunofluorescence analyses showed that ectopic expression of p53 dramatically reversed the expression pattern of the epithelial markers E-cadherin and β-catenin or the mesenchymal markers vimentin and fibronectin in NUMB-knockdown cells (Figure 3A and 3B). Importantly, enhanced expression of p53 also converted the mesenchymal phenotypes of NUMB-knockdown cells into epithelial phenotypes with increased cell-cell adhesion (Figure 3C), without altering cell senescence (Supplementary Figure S2E) and cell growth (Supplementary Figure S2F). These results were further confirmed by applying Nutlin-3, which is an agonist of p53 and is widely used to block p53 degradation [35]. Nutlin-3 treatment completely restored the p53 protein level in NUMB-knockdown MCF10A cells (Figure 3D). Meanwhile, stabilization of p53 by Nutlin-3 treatment completely reversed the expression profiles of EMT-related markers (Figure 3D and 3E) and caused a clear mesenchymal to epithelial switch (Figure 3F) in NUMB-knockdown cells. Re-expression of p53 or stabilization of p53 by Nutlin-3 significantly blocked the increase in the CD44^high^/CD24^low^ stem cell population induced by NUMB silencing (Figure 3G).

To further confirm whether p53 could directly regulate EMT in MCF10A cells, we silenced p53 expression using lentiviral transduction. Similarly to NUMB knockdown, silencing of p53 expression in MCF10A cells converted the epithelial status into mesenchymal status, accompanied by a decrease in the epithelial markers E-cadherin and β-catenin and an increase in the mesenchymal markers vimentin and fibronectin (Supplementary Figure S2G and S2H). Together, these results suggest that knockdown of NUMB promotes EMT by modulating p53 stability in MCF10A cells.

### The negative association between NUMB expression and the EMT program in TNBC cells

**Figure 4:**
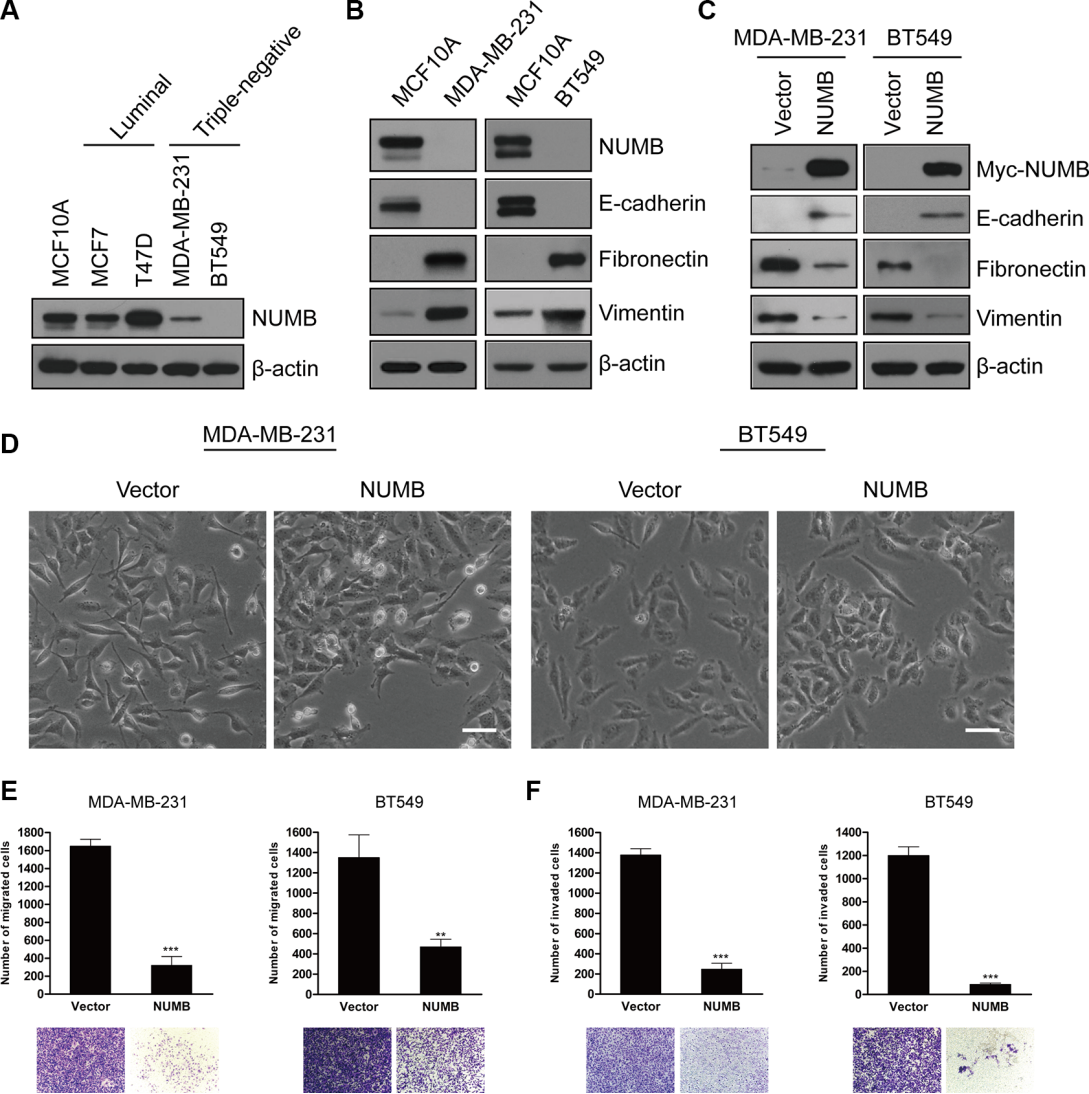
The negative association between NUMB expression and the EMT program in TNBC cells (**A**) Immunoblotting of NUMB in normal MCF10A, luminal (MCF-7 and T47D) and TNBC (MDA-MB-231 and BT549) cell lines. (**B**) Immunoblotting of NUMB and EMT markers in MCF10A, MDA-MB-231 and BT549 cells. (**C**) Immunoblotting of the Myc-tag and EMT markers in MDA-MB-231 and BT549 cells expressing Myc-NUMB or the empty vector. (**D**) Phase-contrast images of MDA-MB-231 and BT549 cells expressing NUMB or the empty vector. Scale bar = 50 μm. (**E**) Migration and (**F**) invasion assays in MDA-MB-231 and BT549 cells expressing NUMB or the empty vector. The mean was derived from cell counts in five fields, and each experiment was repeated three times (***p < 0.001, compared to the control). Representative images of migrated and invaded cells are shown.

To investigate the pathological significance of NUMB-mediated EMT in breast cancer development and progression, we examined the NUMB expression levels in various breast cancer cell lines. Interestingly, among the five examined cell lines, a substantial reduction in NUMB expression was specifically detected in two TNBC cell lines, MDA-MB-231 and BT549 (Figure 4A). Importantly, a negative correlation between NUMB expression and EMT initiation was detected in these two TNBC cell lines (Figure 4B). These data suggest that attenuation of NUMB expression is likely a major cause of EMT initiation during TNBC progression. To further address this issue, we next stably overexpressed NUMB in both MDA-MB-231 and BT549 cells. As expected, NUMB overexpression completely reversed the expression pattern of EMT markers in these TNBC cells (Figure 4C). Moreover, NUMB-expressing cells partially lost their fibroblast-like morphology and displayed tight cell aggregation (Figure 4D). Consistent with these results, the NUMB-expressing TNBC cells showed reduced migration and invasion (Figure 4E and 4F). Combined, these data strongly suggested that the loss of NUMB facilitates EMT initiation in TNBC.

### NUMB negatively regulates EMT in TNBC in a Notch-dependent and p53-independent manner

**Figure 5:**
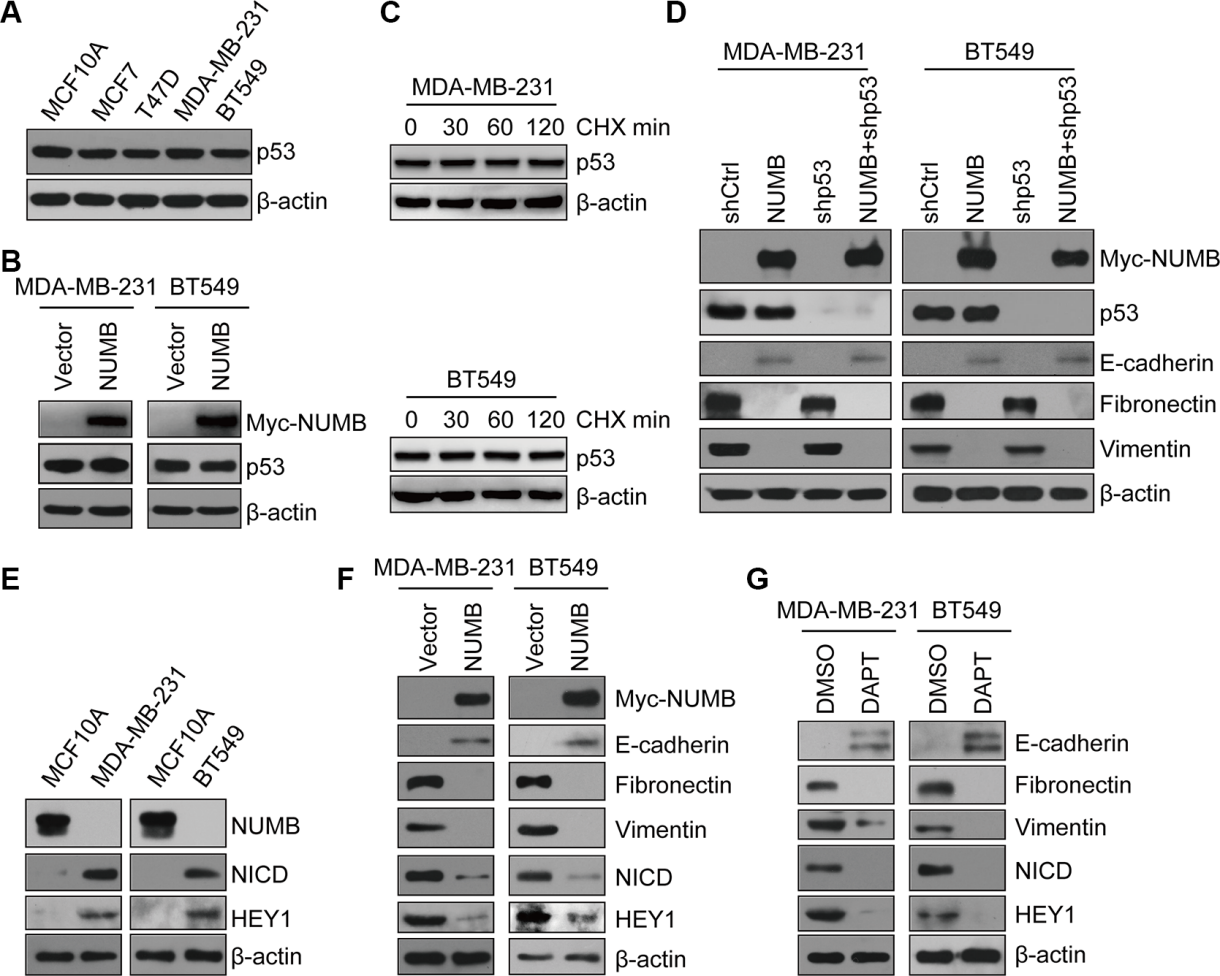
NUMB negatively regulates EMT of TNBC in a Notch-dependent and p53-independent manner (**A**) Immunoblotting of p53 in normal MCF10A, luminal (MCF-7 and T47D) and TNBC (MDA-MB-231 and BT549) cell lines. (**B**) Immunoblotting of NUMB and p53 in MDA-MB-231 and BT549 cells expressing NUMB or the empty vector. (**C**) Immunoblotting of p53 in MDA-MB-231 and BT549 cells treated with cycloheximide (CHX, 100 μg/mL) at the indicated times. (**D**) Expression of NUMB, p53 and EMT markers was analyzed by immunoblotting in MDA-MB-231 and BT549 cells expressing the indicated constructs. (**E**) Immunoblotting of NUMB, NICD and HEY1 in MCF10A, MDA-MB-231 and BT549 cells. (**F**) Immunoblotting of NUMB, NICD, HEY1 and EMT markers in MDA-MB-231 and BT549 cells expressing NUMB or the empty vector. (**G**) Immunoblotting of NUMB, NICD, HEY1 and EMT markers after treatment with DAPT (10 μM) in MDA-MB-231 and BT549 cells.

Having shown that NUMB can regulate EMT in MCF10A cells by stabilizing p53, we then examined the potential correlation between p53 and NUMB expression in various breast cancer cells. However, the p53 protein level was not downregulated in TNBC cells (MDA-MB-231 and BT549) harboring functionally inactive p53 mutants (Figure 5A). Moreover, overexpression of NUMB in TNBC cells had no detectable effect on p53 level (Figure 5B). Mutant p53 protein commonly shows increased stability [36]. We next examined the p53 protein levels in TNBC cells treated with cycloheximide to inhibit new p53 protein. The p53 protein in TNBC cells was relatively stable (Figure 5C). These results indicated that the mutant p53 protein level is not governed by NUMB in TNBC cells. Then, we asked whether p53 signaling contributes to EMT of TNBC cells. For this purpose, p53 was knocked down in the TNBC cell lines MDA-MB-231 and BT549 using lentiviral transduction. Knockdown of p53 did not alter the expression profiles of E-cadherin, fibronectin and vimentin in TNBC cells (Figure 5D). These results indicate that mutant p53 has no function in the regulation of EMT in TNBC cells. To determine whether NUMB contributes to EMT in TNBC in a p53-dependent manner, we then overexpressed NUMB in control and p53-knockdown TNBC cells. As indicated by immunoblotting, NUMB expression resulted in a similar EMT gene expression pattern regardless of whether p53 was present or absent (Figure 5D). Together, these data suggest that the NUMB-mediated negative regulation of EMT in TNBC is independent of mutant p53.

Next, we examined the Notch signaling pathway, which is extensively studied as a critical regulator of the cancer EMT program and is negatively regulated by NUMB during asymmetric cell division [25, 37]. We found that the expression levels of the Notch1 intracellular domain (NICD) and HEY1 were dramatically increased in both MDA-MB-231 cells and BT549 cells compared with MCF10A cells, indicating that the Notch signaling is highly activated in these TNBC cell lines (Figure 5E and Supplementary Figure S3). We speculated that the activation of Notch signaling in TNBC cells is due to the substantial reduction in NUMB expression. To test this hypothesis, we overexpressed NUMB in these two TNBC cell lines and assessed the Notch activity in these cells. NUMB overexpression efficiently decreased the levels of NICD and HEY1 in both MDA-MB-231 cells and BT549 cells (Figure 5F). In parallel with this decline in Notch activity, NUMB overexpression also resulted in an upregulation of E-cadherin and downregulation of vimentin and fibronectin in MDA-MB-231 and BT549 cells (Figure 5F). Thus, these data strongly suggest that loss of NUMB promoted a positive correlation between Notch activity and EMT generation in TNBC cells. To further confirm the significance of Notch signaling during EMT in TNBC, DAPT, a γ-secretase inhibitor, was applied to block Notch signaling in TNBC cells. Both NICD and HEY1 expression was efficiently reduced following DAPT treatment. Meanwhile, inhibition of Notch activity by DAPT treatment caused obvious EMT suppression in both examined TNBC cell lines (Figure 5G), indicating that Notch signaling was required for EMT of TNBC. Based on these data, we propose that the suppression of NUMB-mediated activation of Notch signaling contributes to the EMT program in TNBC cells.

### NUMB negatively regulates EMT in breast cancer cells expressing wild-type p53 in a p53-dependent manner

**Figure 6:**
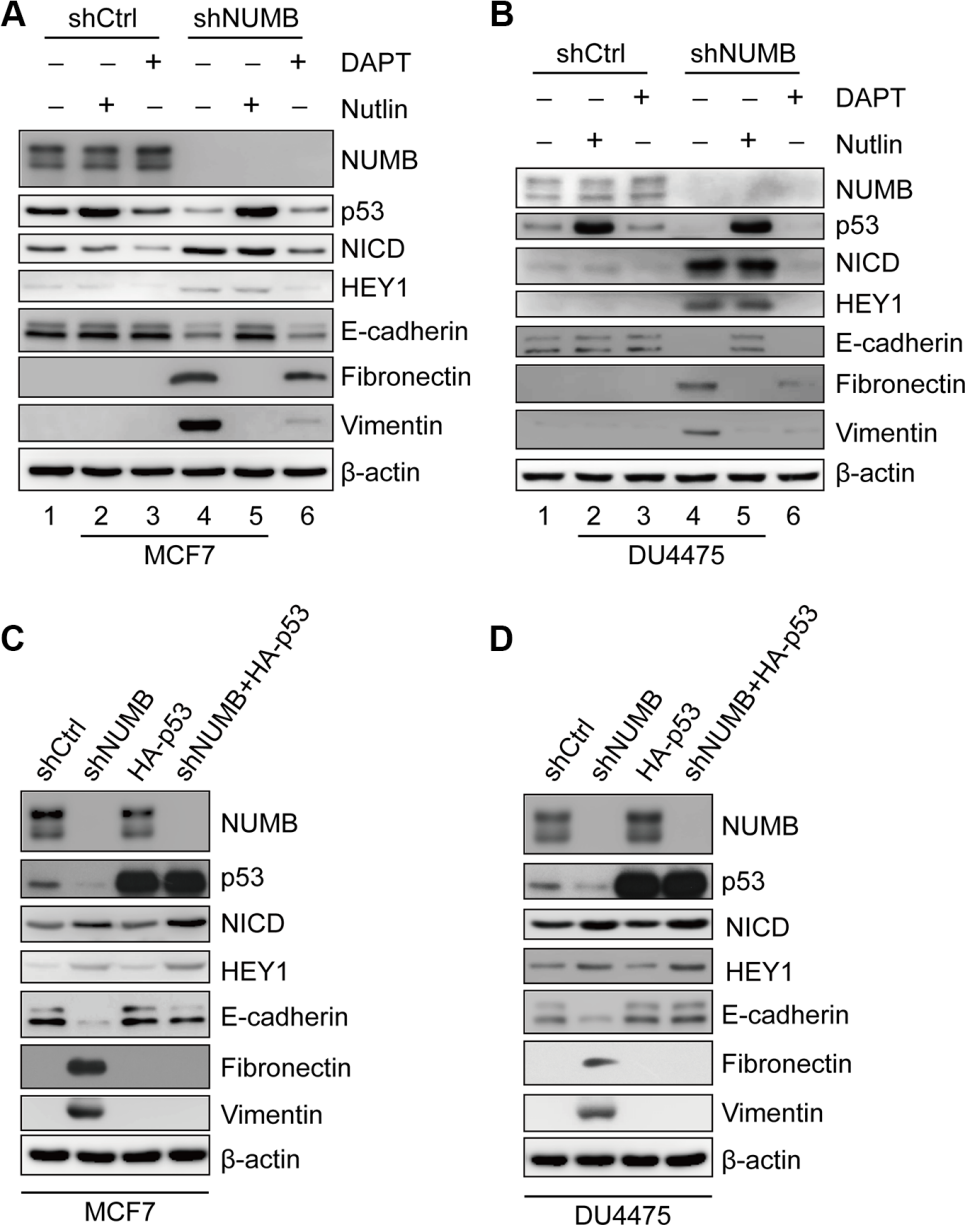
The p53 pathway plays a major role in NUMB-mediated EMT in breast cancer cells expressing wild-type p53 (**A**) Immunoblotting of NUMB, p53, NICD, HEY1 and EMT markers after treatment with Nutlin-3 (10 μM) or DAPT (10 μM) in MCF7 cells with shCtrl and shNUMB. (**B**) Immunoblotting of NUMB, p53, NICD, HEY1 and EMT markers after treatment with Nutlin-3 (10 μM) or DAPT (10 μM) in DU4475 cells with shCtrl and shNUMB. (**C**) Expression of NUMB, p53, NICD, HEY1 and EMT markers was analyzed by immunoblotting in MCF7 cells expressing the indicated constructs. (**D**) Expression of NUMB, p53, NICD, HEY1 and EMT markers was analyzed by immunoblotting in DU4475 cells expressing the indicated constructs.

We showed that NUMB can regulate EMT in MCF10A cells by stabilizing wild-type p53, while in TNBC cells with mutant p53, regulation occurs via Notch signaling. We next investigated the pathway employed by NUMB to regulate EMT in breast cancer cells with wild-type p53. Thus, we used the luminal breast cancer cell line MCF7 and the TNBC cell line DU4475, both of which express wild-type p53. After NUMB knockdown, p53 protein levels decreased, while expression of NICD and HEY1 increased (Figure 6A and 6B, lane 1 vs lane 4). Meanwhile, NUMB knockdown also resulted in downregulation of E-cadherin and upregulation of vimentin and fibronectin in the MCF7 and DU4475 cells (Figure 6A and 6B, lane 1 vs lane 4). These results suggest that suppression of NUMB decreased the p53 protein level and triggered the EMT process in breast cancer cells expressing wild-type p53. Then, we treated NUMB-knockdown cells with Nutlin-3 to block p53 degradation. Stabilization of p53 by Nutlin-3 treatment completely reversed the expression profiles of the EMT-related markers (Figure 6A and 6B, lane 4 vs lane 5), but Nutlin-3 had no significant effect on Notch activity in NUMB-knockdown cells (Figure 6A and 6B, lane 4 vs lane 5). These results indicate that p53 completely rescues the NUMB knockdown-mediated EMT phenotype independent of Notch signaling. We further used DAPT to abolish Notch activity in NUMB-knockdown cells. We observed that DAPT had no significant effect on p53 levels in NUMB-knockdown or control cells (Figure 6A and 6B, lane 4 vs lane 6 and lane 1 vs lane 3). Meanwhile, Notch inactivation did not rescue the expression of E-cadherin, a hallmark of EMT (Figure 6A and 6B, lane 4 vs lane 6), and it only resulted in slightly reduced expression of fibronectin and vimentin (Figure 6A and 6B, lane 4 vs lane 6) in NUMB-knockdown cells. These results indicate that in the case of p53 downregulation, Notch inactivation only slightly influenced the expression of mesenchymal markers in NUMB-knockdown cells. In addition, neither Nutlin-3 nor DAPT treatment had any effect on the EMT marker expression profile in control cells (Figure 6A and 6B, lane 1, 2 and 3). This may be due to the existence of an epithelial-like protein expression pattern with high levels of E-cadherin and low levels of fibronectin and vimentin in the two cell lines. Combined, these results imply that the p53 pathway plays a major role in NUMB-mediated EMT in breast cancer cells with wild-type p53. To further confirm that reduced p53 expression is responsible for NUMB knockdown-induced EMT initiation, we exogenously expressed p53 in NUMB-knockdown breast cancer cells with wild-type p53 through lentiviral transduction. As shown in Figure 6C and 6D, enhanced expression of p53 reversed the expression pattern of either epithelial markers, such as E-cadherin, or mesenchymal markers, such as vimentin and fibronectin, in NUMB-knockdown breast cancer cells; however, it had no significant effect on the expression of NICD and HEY1 in NUMB-knockdown breast cancer cells. Taken together, the p53 pathway appears to be a primary regulator of NUMB-mediated EMT in breast cancer cells with wild-type p53.

NUMB loss or low expression is frequently detected in breast cancer [38]. We next investigated whether these tumors harbored reduced p53 levels and impaired p53-mediated responses. To address this issue, we compared the expression of NUMB and p53 in the breast cancer cell lines MCF7 and DU4475. We observed that the levels of p53 in DU4475 cells were lower than those in MCF7 cells (Supplementary Figure S4). Correspondingly, DU4475 cells with low p53 levels also had lower expression of NUMB compared to that of the MCF7 cells (Supplementary Figure S4). Moreover, we found that in DU4475 cells, the stability of p53 was reduced due to increased proteasomal degradation compared with that of MCF7 cells, and restoration of p53 was observed in DU4475 cells following treatment with the proteasome inhibitor MG132 (Supplementary Figure S4). These data suggest that loss of NUMB also resulted in instability of p53 in breast cancer cells with wild-type p53.

### NUMB overexpression decreases tumor growth and metastasis of TNBC cells *in vivo*

**Figure 7:**
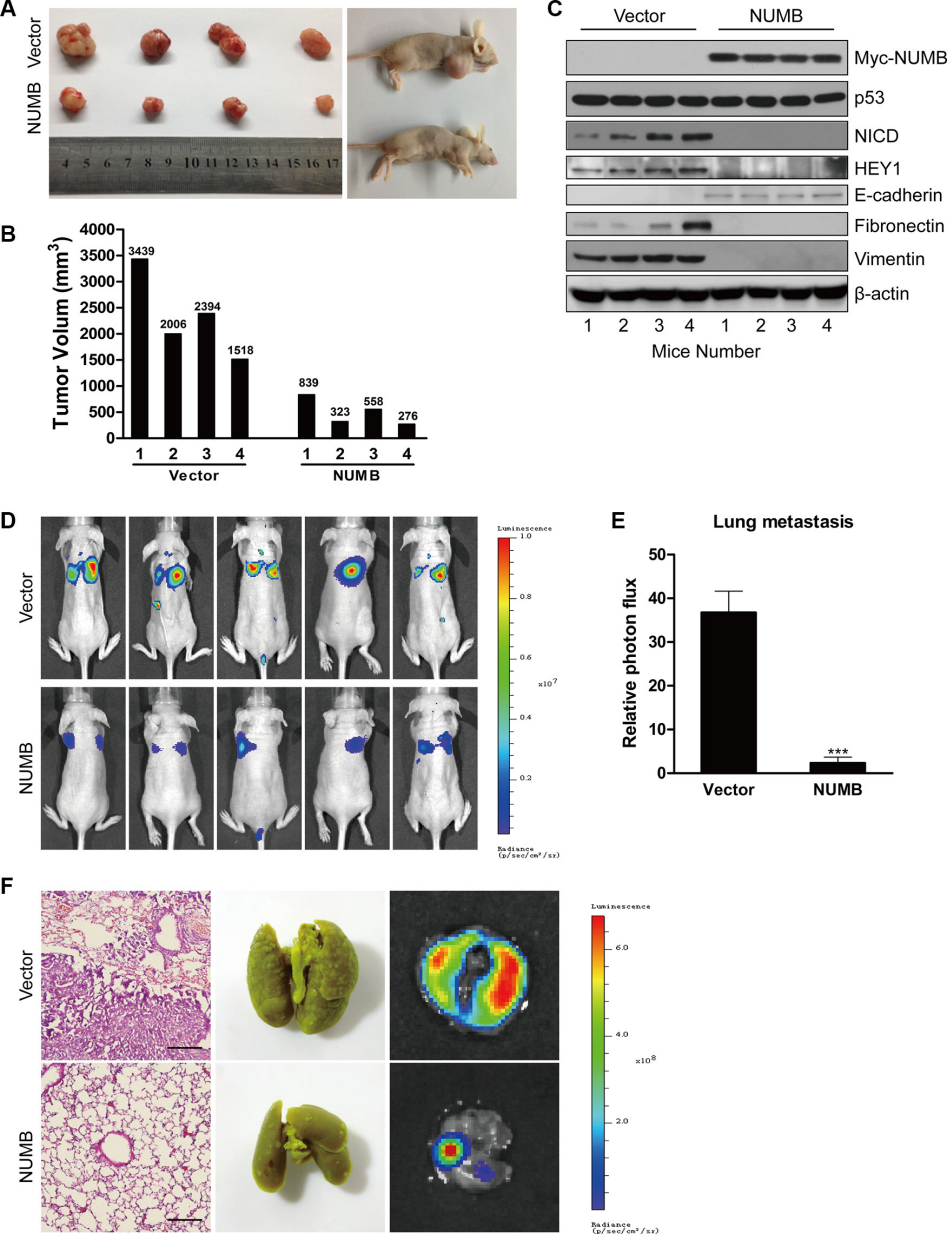
NUMB overexpression suppresses tumor growth and metastasis of TNBC cells *in vivo* (**A**) MDA-MB-231 cells expressing NUMB or the empty vector were subcutaneously injected into BALB/c female nude mice separately (*n* = 4). Photographs of representative tumors are shown. (**B**) Individual tumor volume was measured at week 10 after subcutaneous injection of MDA-MB-231 cells expressing NUMB or the empty vector. (**C**) Expression of NUMB, p53, NICD, HEY1 and EMT markers was analyzed by immunoblotting of mouse tumors derived from NUMB or empty vector-carrying cells. (**D**) MDA-MB-231-Luc cells transduced with NUMB or the empty vector were injected into the tail vein of BALB/c female nude mice (*n* = 5), and the metastases were measured by bioluminescence 10 weeks after the initial injection. Representative *in vivo* bioluminescence images are shown. (**E**) The above-described breast cancer metastases to the lung were quantified using bioluminescence imaging (***p < 0.001, compared to the control). (**F**) H&E staining (left), morphology (middle) and *in vitro* bioluminescence measurement (right) of lung metastases isolated from the above-described nude mice. Scale bar =100 μm.

To determine the role of NUMB in breast tumorigenesis *in vivo*, we generated a xenograft mouse model by subcutaneous injection of MDA-MB-231 cells overexpressing NUMB and their corresponding control cells into female nude mice, and the growth of the tumors was measured at 10 week post-implantation. As shown in Figure 7A and 7B, NUMB overexpression resulted in a dramatic reduction in tumor volume, suggesting that NUMB significantly decreased tumor incidence and growth in the breast cancer xenograft model. To further test whether NUMB inhibits tumor growth of TNBC by blocking the Notch pathway *in vivo*, mouse tumors derived from vector and NUMB-expressing cells were analyzed using immunoblotting. As shown in Figure 7C, both NICD and HEY1 were efficiently reduced in tumors overexpressing NUMB. Additionally, expression of the mesenchymal markers fibronectin and vimentin was dramatically downregulated, and the epithelial marker E-cadherin was dramatically upregulated in tumors overexpressing NUMB. However, no appreciable change in the expression of p53 was found in tumors of mice carrying vector and NUMB-expressing cells. Thus, these data suggest that NUMB mediates tumor inhibition of TNBC via blocking Notch signaling but not the p53 pathway.

We next tested whether NUMB could also inhibit tumor metastasis *in vivo*. For this purpose, MDA-MB-231 cells that stably expressed firefly luciferase (MDA-MB-231-Luc) were infected with lentiviruses carrying NUMB. NUMB-expressing MDA-MB-231-Luc or control cells were then injected into the tail veins of nude mice, and the lungs were examined for metastases by bioluminescence and quantified at 10 weeks after injection (Figure 7D and 7E). The results showed that the bioluminescence signal in the lung was strikingly decreased in the mice injected with NUMB-expressing cells, indicating that NUMB negatively regulated the metastasis of breast cancer cells to the lung. The metastases to the lungs were verified by H&E staining, lung metastasis morphology and bioluminescence imaging (Figure 7F). Taken together, these findings demonstrate the inhibitory effects of NUMB in breast cancer progression and metastasis.

### High NUMB levels delineate breast cancer patients with good clinical outcomes

**Figure 8:**
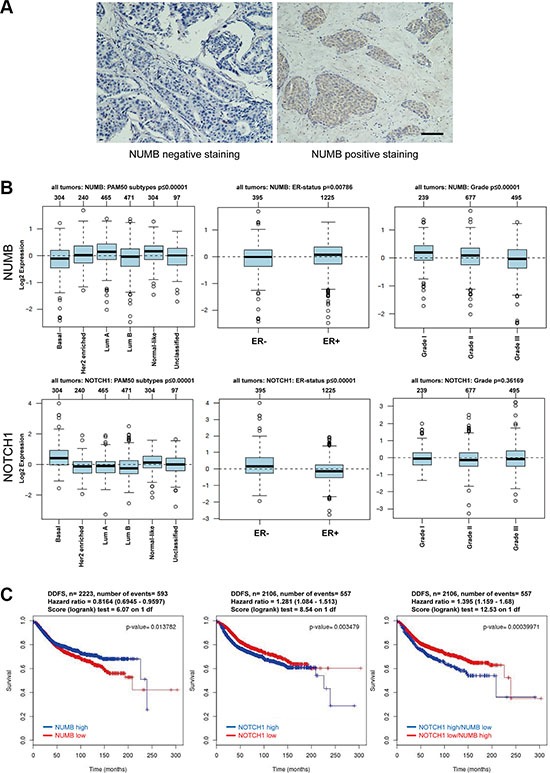
Low expression of NUMB is associated with poor prognosis in breast cancer (**A**) Representative images of the immunohistochemical staining of NUMB. Negative expression of NUMB protein (left). Positive expression of NUMB protein (right). Scale bar =100 μm. (**B**) Analysis of expression of NUMB (upper) and Notch1 (lower) in breast cancer tumors with different molecular subtypes (left, *n* = 1280, NUMB is low in the BLBC subtype; *p* < 0.00001, whereas Notch1 is high in the BLBC; *p* < 0.00001), ER status (middle, *n* = 1620, NUMB is high in ER-positive breast cancer; *p* = 0.00786, whereas Notch1 is high in ER-negative breast cancer; *p* < 0.00001) and pathological grades (right, *n* = 1411, NUMB decreases progressively from grade 1 to grade 3; *p* < 0.00001, whereas Notch1 expression has no statistical significance from grade 1 to grade 3; *p* = 0.36169). (**C**) Kaplan-Meier survival analysis of the publicly available datasets for evaluating the effects of NUMB and Notch1 expression on distant disease-free survival (DDFS) of breast cancer patients.

Considering the *in vitro* and *in vivo* data, we investigated whether NUMB expression is clinically correlated with human breast cancer progression. We expanded our observations using immunohistochemistry staining of NUMB in 150 human breast tumor samples. The results showed that NUMB protein levels were lower in the subset of breast cancer samples with poor prognostic parameters, such as ER-negative (*p* < 0.001), HER2-positive (*p* = 0.016) and triple-negative (*p* = 0.023) phenotypes (Figure 8A and Table 1). Kaplan-Meier survival analysis using the publicly available datasets [39] demonstrated that higher NUMB expression was associated with a better overall survival (OS, *p* = 0.036) and disease-free survival (DFS, *p* = 0.028) of breast cancer patients (Supplementary Figure S5). These observations demonstrate that high NUMB expression was significantly correlated with breast cancer patients with good clinical outcomes.

**Table 1:** Association between NUMB expression and tumor subtypes in invasive ductal carcinomas of breast

Tumor type	Total no. of cases	No. with positive NUMB staining	Percentage with positive NUMB staining (%)	*P* value
Estrogen receptor	150			< 0.001
positive	71	57	80.3	
negative	79	39	49.4	
ERBB/HER2	150			0.016
positive	36	17	47.2	
negative	114	79	69.3	
Triple-negative	150			0.023
Yes	41	20	48.8	
No	109	75	68.8	

To further investigate the clinicopathological relevance of the NUMB/Notch axis in breast cancer progression, we expanded our observations using publicly available mRNA profiling datasets of breast cancer patients [40]. We respectively evaluated the expression of NUMB and Notch1 in different breast cancer subtypes, ER status and tumor grade. The results showed that NUMB was differentially expressed in different subtypes of breast cancer based on the PAM50 gene signature, with relative high expression in luminal A and low expression in basal-like breast cancer (BLBC) subtypes (*n* = 1881, *p* < 0.00001) (Figure 8B, upper left), which shares many clinicopathological and molecular features with TNBC [2, 8, 11]. We also observed that NUMB expression was lower in the ER-negative breast cancer patients compared to the ER-positive breast cancer patients (*n* = 1620, *p* = 0.00786) (Figure 8B, upper middle). Furthermore, evaluation of NUMB mRNA expression in different tumor histological grades showed that its expression was higher in grade 1 and lower in grade 3 tumors (*n* = 1411, *p* < 0.00001) (Figure 8B, upper right). In contrast to the low expression of NUMB in BLBC and ER-negative breast cancer patients, Notch1 had a relatively high expression in these breast cancer patients, demonstrating an apparent opposite trend for the expression of NUMB and Notch1 in breast cancers (Figure 8B, lower left and middle). This implies that low NUMB expression and high Notch1 expression in BLBC may be involved in a potential role for NUMB-mediated control of Notch signaling in this subtype. We further expanded our analysis to other EMT regulators. Similar to NUMB, the EMT negative regulators GATA3 and FOXA1 had significantly lower expression in the BLBC subtypes, ER-negative and higher histological grade breast cancer patients, while the EMT inducers FOXC1, FOXC2 and SNAI1 mimicked the expression profiles of Notch1 (Supplementary Figure S6). These results imply a clinical correlation among NUMB, Notch, EMT and TNBC. Moreover, Kaplan-Meier survival analysis [39] revealed that high NUMB expression was associated with decreased distant disease-free survival (DDFS *p* = 0.0138) of breast cancer patients (Figure 8C, left), while high Notch1 expression was associated with increased DDFS (*p* = 0.0035) of breast cancer patients (Figure 8C, middle). These results indicate that low NUMB levels or high Notch1 levels are associated with distant metastasis of breast cancer. Further stratification of patient groups based on inverse Notch1/NUMB expression improved the predictive capability of NUMB for DDFS (*p* = 0.0004) in breast cancer patients (Figure 8C, right), suggesting that NUMB may be another significant predictor of distant metastasis. Taken together, these clinical data implicate the NUMB/Notch axis in the regulation of EMT-mediated TNBC metastasis.

## DISCUSSION

NUMB has been linked to human cancers as a tumor suppressor. The expression of NUMB is frequently lost in several human cancers, including leukemia and lung and brain cancers [21, 41, 42]. Loss of NUMB expression destabilizes p53 and enhances proliferation and chemoresistance in breast cancer [20]. More recently, it was reported that phosphorylation of NUMB by aPKCζ promotes liver tumorigenesis by destabilizing p53 [32]. Moreover, loss of NUMB expression can confer a proliferative advantage on breast cancer and non-small cell lung cancer by antagonizing Notch [21, 38]. Based on these studies, NUMB has been proposed as a potential therapeutic target for tumorigenesis in several cancers, such as prostate, liver, breast and lung cancers [21, 32, 38, 43]. In this study, we are unique in demonstrating that NUMB is a negative regulator of EMT, and reduced NUMB expression facilitates TNBC metastasis by inhibiting Notch activity.

Previous studies have shown that NUMB is a crucial regulator of EMT in mammalian epithelial cells [14, 23, 44]. Several reports further indicate that NUMB may be involved in EMT in cancer [45, 46]. However, the underlying mechanism by which NUMB regulates EMT in cancer cells remains largely unknown. Colaluca and colleagues reported that NUMB could stabilize p53 by inhibiting the MDM2-mediated ubiquitination of p53 [20]. More recently, it was reported that dysfunction of the NUMB/p53 axis in primary mammary epithelial cell leads to a stem cell-like phenotype via EMT-dependent dedifferentiation and ultimately results in tumorigenesis [45]. Here, we demonstrate that loss of NUMB promotes EMT through the direct regulation of p53 stability in normal human mammary epithelial cells and breast cancer cells expressing wild-type p53. The emerging evidence demonstrates that p53 plays a pivotal role in opposing EMT and that loss of this antagonism may contribute to the induction of an EMT-like phenotype in p53-null tumors [24, 34]. Loss or inhibition of p53 function has been reported to promote EMT by regulating miR-34 and miR-200c in several normal and cancer cell lines with wild-type p53 [24, 34, 47]. Shiota et al. showed that Twist inhibits wild-type p53 function via a direct interaction with p53 [48]. Twist is believed to antagonize p53 function to promote EMT, indicating that p53 maintains a transcriptional program to prevent EMT. Apparently, the expression of NUMB is downregulated, leading to reduced expression of p53. This, in turn, abolishes the p53-mediated suppression of EMT, eventually resulting in induction of EMT in normal mammary cells and breast cancer cells with wild-type p53.

Interestingly, our study further revealed a negative correlation between NUMB expression and EMT in TNBC cells, suggesting that loss of NUMB might contribute to EMT in TNBC. Recent comprehensive molecular analyses demonstrated that approximate 85% of TNBC harbor p53 mutations [49]. Mutant p53 protein is intrinsically stable and is not regulated by NUMB. Correspondingly, our results show that NUMB-mediated negative regulation of EMT in TNBC cells is p53 independent. This may be because p53 mutations lead to the loss of wild-type p53 activity. It has become increasingly clear that the mutant p53 protein may not only result in loss of wild-type p53 tumor suppressor activity, but may also gain new oncogenic properties [50]. Indeed, in our hands, knockdown of mutated p53 did not alter the expression of EMT markers in TNBC cells, which is consistent with a previous report in BT549 cells [24]. Interestingly, it has been reported that mutant p53 is able to trigger a partial EMT-like transition in some cells [51, 52]. Based on these data, we hypothesized that mutant p53 may be sufficient but not necessary for maintaining the EMT phenotype. Our clinical data showed that Notch signaling is commonly activated in TNBC. Meanwhile, we found that Notch signaling is highly activated in TNBC cells with mutant p53, and overexpression of NUMB suppressed the Notch activation as well as EMT in TNBC cells. These data strongly suggest that NUMB acts as a negative regulator of EMT in TNBC by antagonizing Notch signaling but not mutant p53. Apparently, loss of function of NUMB in TNBC activates the Notch pathway, which further maintains the mesenchymal phenotype of TNBC.

Our data demonstrated that NUMB negatively regulates the EMT process in normal mammary epithelial cells and breast cancers. Interestingly, Wu et al. reported that conditional deletion of NUMB and NUMB-like in epicardial cells causes epicardial cell EMT defects [53]. EMT is classified into three subtypes based on the biological context in which they occur [54]. While these three classes of EMTs represent distinct biological processes, distinct types of EMT use different initiation signals, transcription factors and signaling pathways. The distinct role of NUMB in EMT in different physiological process highlights the importance of cell context and genetic background in development and cancer EMT processes.

TNBC is more aggressive and difficult to treat because of the lack of effective therapeutic targets. Recent studies have linked EMT with the aggressive BLBC [8]. Our results demonstrated that NUMB overexpression in TNBC cell lines reverses the mesenchymal phenotype and inhibits metastasis *in vivo*. Furthermore, clinical data showed that NUMB expression is lower, while Notch1 expression is higher in TNBC. Kaplan-Meier survival analysis showed that inverse Notch1/NUMB expression is a strong predictor of DDFS in breast cancer. This implies that low NUMB expression and high Notch1 expression in TNBC may be involved in a potential role for NUMB-mediated antagonism of Notch signaling in this subtype. As noted in a recent review, NUMB inhibition of Notch activity may serve as a therapeutic target in prostate cancer [43]. Our work on NUMB adds to the growing body of evidence indicating that targeting the EMT-like phenotypes represents a potential strategy for the development of novel anticancer therapeutics for TNBC. We also analyzed other EMT regulators based on breast cancer datasets. Similar to NUMB, the EMT negative regulators GATA3 and FOXA1 had significantly lower expression in the BLBC subtypes and in ER-negative and higher histological grade breast cancer patients, whereas the EMT inducers FOXC1, FOXC2 and SNAI1 mimicked the expression profile of Notch1. These results not only suggest interactions among these EMT regulators in clinical breast cancer samples but also indicate the pathological relevance of inverse Notch1/NUMB expression in the regulation of EMT and TNBC progression. Apparently, a negative correlation between NUMB and Notch1 exists in which loss of function of NUMB in TNBC leads to elevated expression of Notch1, which, in turn, further promotes EMT and eventually leads to the metastasis of TNBC. Our results in this study suggest the possibility of using NUMB as a novel biomarker for prognosis and diagnosis of TNBC, as well as a potential candidate for targeted therapy of this highly aggressive breast cancer.

## MATERIALS AND METHODS

### Antibodies and reagents

Antibodies against E-cadherin, β-catenin, Fibronectin, Vimentin (BD Biosciences), NUMB, Notch1, Cleaved Notch1 (NICD), Oct4 and SOX2 (Cell Signaling Technology), β-actin (Santa Cruz), HEY1 (Millipore), Myc-tag, HA-tag (Biolegend) and p53 (Sigma) were used. Recombinant human hEGF and hFGF proteins were purchased from R&D Systems. B27 and horse serum were obtained from Gibico. Insulin, Cholera toxin, hydrocortisone, MG132 and Nutlin-3 were purchased from Sigma.

### Cell culture

MCF10A, MCF7, T47D, DU4475, MDA-MB-231, BT549, and 293T cell lines were obtained from the American Type Culture Collection. MCF10A cells were cultured as previously described [55] in DMEM/F12 supplemented with 5% horse serum, 20 ng/mL EGF, 0.5 mg/mL hydrocortisone, 100 ng/mL cholera toxin, 10 mg/mL insulin and pen/strep. MCF7, MDA-MB-231 and 293T cells were cultured in DMEM containing 10% FBS (Corning). T47D, DU4475 and BT549 cells were cultured in RPMI-1640 medium supplemented with 10% FBS.

### Lentiviral production and infection of target cells

The recombinant lentiviruses expressing Myc-NUMB, p53, shNUMB#1, shNUMB#2 and shp53 were produced by co-transfecting 293T cells with one of the expression plasmids and packaging plasmids (psPAX2 and pMD2.G). Viral supernatants were collected 48 h later, centrifuged to remove cell debris, filtered through 0.45-μm filters (Millipore) and concentrated using Amicon Ultra centrifugal filters (Millipore 100KD MWCO). The cells were transduced with the lentivirus for 12 h in the presence of polybrene (8 μg/mL). Targeting sequences of shRNA are as follows: shNUMB#1: ATACATAGCCATAATGATTGC, shNU MB#2: TTACATTTCAGTAAATGTG, shp53: GACTC CAGTGGTAATCTACT.

### Immunoblot analysis

Cells were lysed in RIPA lysis buffer (Applygen) containing broad-spectrum halt protease and phosphatase inhibitor cocktail (Pierce). Protein lysates were resolved by SDS-PAGE, transferred to PVDF membranes, detected with primary antibody overnight at 4°C, and then incubated with HRP-conjugated secondary antibodies. Immunoblots were visualized with ECL reagent (Millipore).

### Immunofluorescence

Cells were seeded onto glass coverslips in 24-well plates, washed with PBS, fixed in 4% formaldehyde solution and permeabilized with 0.2% Triton X-100/PBS. Cells were blocked with 2% BSA in PBS for 30 min. Coverslips were incubated with primary antibodies for 1 h, followed by incubation with FITC-conjugated secondary antibodies for 1 h, and then stained with DAPI. Finally, coverslips were observed under a fluorescence microscope.

### Immunohistochemistry

Immunohistochemical analysis of NUMB was performed on human breast cancer tissue microarrays (Alenabio). The tissue array sections were deparaffinized and rehydrated. Antigen retrieval was done by heating the sample in citrate buffer (Beyotime) at 95°C for 15 minutes. Endogenous peroxidase activity was blocked with 3% hydrogen peroxide, then sections were blocked with 10% goat serum. Sections were incubated with anti-NUMB antibody (1:50) for 2 hours, followed by incubation with secondary antibodies for 1 hour. The immunostaining was developed with 3.30-diaminobenzidine (Sigma). Finally, sections were counterstained with hematoxylin. The extent of NUMB immunoreactivity was scored as: negative (−) for less than 5% positive cells, low (+) for weak or sparse (5%–25%) staining, moderate (++) for intermediate (25–50%) staining and high (+++) for strong and intense (> 50%) staining separately in human breast tumor specimens. Clinical information of breast cancer specimens such as tumor size and metastatic status, tumor grade, tumor stage and status of ER, PR & Her2 was available. All human tissues are collected under IRB and HIPPA approved protocols. All samples have been tested negative for HIV and Hepatitis B.

### Mammosphere formation assay

Mammosphere assay was performed as described [56] with minor modifications. Single cells were plated at 10,000 cells/mL on 6-well ultra-low attachment plates (Corning) in serum-free DMEM/F12 medium supplemented with 20 ng/mL bFGF, 20 ng/mL EGF, 4 μg/mL insulin, 4 μg/mL heparin, 1 μg/mL hydrocortisone, 0.4% BSA and B27. Fresh medium was supplemented every three days. After 14 d of incubation, the mammospheres were counted.

### Flow cytometry

1 × 10^6^ cells were resuspended in 100 μL PBS containing 2% FBS (FACS buffer), and then incubated on ice for 10 min. CD44-APC and CD24-PE (BD Biosciences) were added to the cell suspension and incubated on ice for 30 min. Cells were washed and resuspended in 500 μL FACS buffer and analyzed using a FACS Calibur Flow Cytometer (BD Biosciences).

### Transwell migration and invasion assays

*In vitro* cell migration and invasion assays were performed using transwell chambers with polyethylene terephthalate membrane (24-well inserts, 8.0 μm; Corning). For the migration assay, 2.5 × 10^4^ cells were added to the top chambers. For the invasion assay, 5 × 10^4^ cells were seeded into the top chamber coated with Matrigel (BD Biosciences). Complete medium was added to the bottom wells to stimulate migration or invasion. After cells were incubated for 24–48 h, they were stained with 0.1% Crystal Violet. Five fields per filter were counted using Image-pro plus software.

### RT-PCR and quantitative PCR

Total RNA was extracted using Trizol reagent (Transgen), according to the manufacturer's instructions. RT-PCR was performed using the Access RT-PCR System from Promega. Quantitative PCR (qPCR) was done using SYBR Green Supermix (Bio-Rad) on a CFX96™ Real Time PCR system (Bio-Rad). GAPDH was used as an internal control in all the experiments. The sequences of PCR primers are listed as follows: NUMB: forward, 5′-ACTTTTGATGCTAGTCGGACC-3′; reverse, 5′-GAAGTAGGAGAGGTGGGAGAG-3′. p53: forward, 5′-CCTCCTCAGCATCTTATCCG-3′; reverse, 5′-CACAAACACGCACCTCAAA-3′. GAPDH: forward, 5′-ACCCAGAAGACTGTGGATGG-3′; reverse, 5′-TTCAGCTCAGGGATGACCTT-3′.

### Xenograft mouse experiments

1 × 10^7^ cells in 100 μL of PBS were injected subcutaneously into 4-week-old female BALB/c nude mice. Four mice per group were used in each experiment. Tumor volume was measured weekly using a vernier caliper and calculated according to the formula: π/6 × length × width^2^. Ten weeks later, the mice were sacrificed, and tumors were collected and photographed. All animal experiments were approved by the Animal Care Committee of the Shenzhen Institutes of Advanced Technology, Chinese Academy of Sciences.

### *In vivo* metastasis assay

MDA-MB-231 cells stably expressing firefly luciferase (MDA-MB-231-Luc) were infected with lentiviruses carrying empty vector or NUMB expression construct. These cells were injected into the lateral tail vein (3 × 10^6^ cells) of 4-week-old female nude mice (five mice per group). Ten weeks later, for bioluminescence imaging, mice were intraperitoneally injected with 150 mg/g of D-luciferin (Goldbio) in PBS. At 10 min after injection, mice were anesthetized and bioluminescence was imaged with an IVIS imaging system and analyzed with Living Image software (Xenogen). All animal experiments were approved by the Animal Care Committee of the Shenzhen Institutes of Advanced Technology, Chinese Academy of Sciences.

### Cell proliferation assays

Cell growth rates were assessed using the Cell Counting Kit-8 (CCK-8, Transgen). Cells were seeded onto 96-well plates with 100 μL suspension per well (2 × 10^3^ cells/well) in triplicate. After incubation for indicated time, CCK-8 solution was added to each well and incubated for 4 h at 37°C. Cell viability was determined by measuring the absorbance at 450 nm.

### Senescence-associated β-galactosidase (SA-β-gal) staining

SA-β-gal staining was done with a senescence-associated β-Galactosidase Staining Kit (Beyotime). Cells were washed with PBS and fixed with 4% paraformaldehyde for 15 min. Next, the cells were stained with X-gal solution overnight at 37°C in darkness. Finally, photographs were taken using an inverted microscope.

### Statistical analysis

Data are presented as mean ± SD. The Student's *t* test (two-tailed) was used to determine statistically the significance of differences between groups. *P* < 0.05 was considered statistically significant. NUMB expression intensities in human breast cancer samples were analyzed by χ^2^ test. Statistical analysis was carried out using the SPSS17.0 software.

## SUPPLEMENTARY MATERIALS FIGURES


