# Supplementary material for: NUMB negatively regulates the epithelial-mesenchymal transition of triple-negative breast cancer by antagonizing Notch signaling
Source: Oncotarget. 2016 Aug 5;7(38):61036–53. doi: 10.18632/oncotarget.11062 (PMC5308634; doi:10.18632/oncotarget.11062)
Supplement: Supplementary file 1 [file oncotarget-07-61036-s001.pdf]

NUMB negatively regulates the epithelial-mesenchymal transition of triple-negative breast cancer by antagonizing Notch signaling

Supplementary Materials

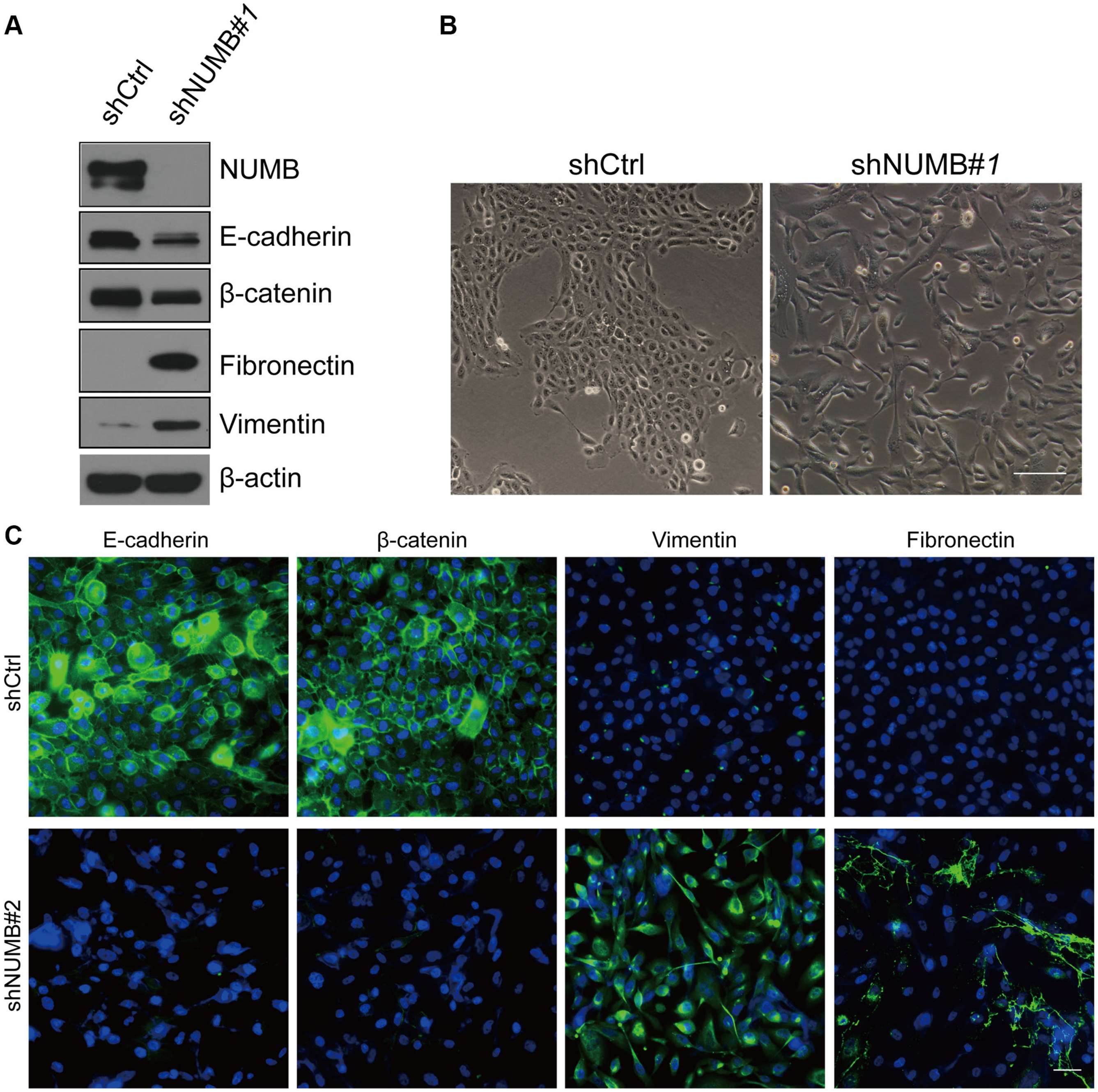

Supplementary Figure S1: NUMB knockdown induces EMT in MCF10A cells. (**A**) Immunoblotting of NUMB and EMT markers in MCF10A cells with shCtrl and shNUMB#1. (**B**) Morphology of MCF10A cells with shCtrl and shNUMB#1. Scale bar =100 μm. (**C**) Immunofluorescence staining of the EMT markers at high cell density. Scale bar = 100 μm.

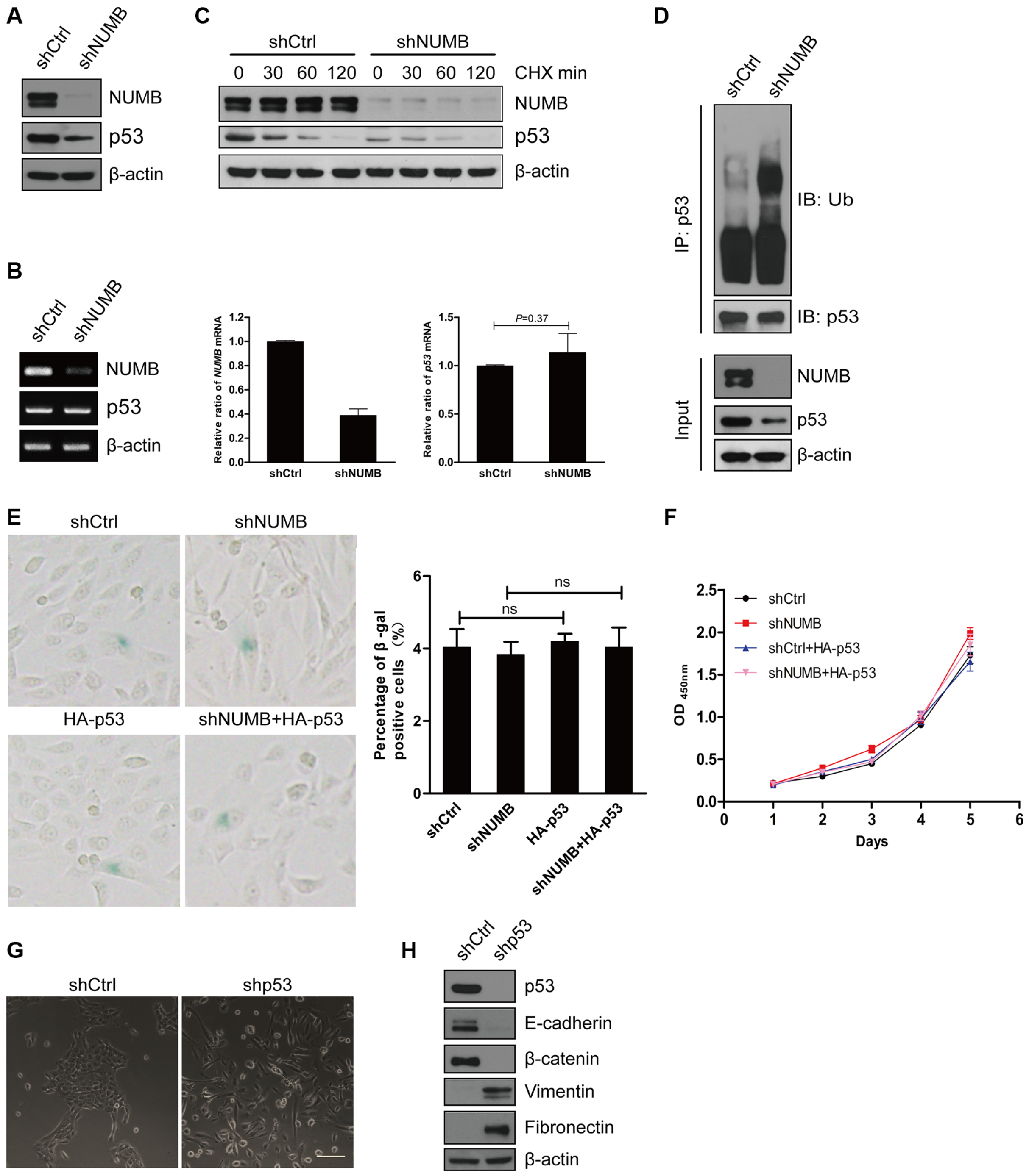

Supplementary Figure S2: NUMB stabilizes p53 in MCF10A cells. (**A**) Immunoblotting analysis of the p53 protein expression in MCF10A cells after NUMB silencing. (**B**) The levels of p53 and NUMB mRNA expression in MCF10A cells after NUMB silencing were measured by RT-PCR (left) and qPCR (middle and right). (**C**) Immunoblotting of NUMB and p53 in MCF10A cells expressing shCtrl and shNUMB treated with cycloheximide (CHX, 100 μg/mL) at indicated times. (**D**) Endogenous p53 was immunoprecipitated and levels of ubiquitination were assessed by immunoblotting in MCF10A cells after NUMB silencing. (**E**) β-gal senescence assay in MCF10A cells expressing the indicated constructs. (**F**) CCK-8 cell growth assay in MCF10A cells expressing the indicated constructs. (**G**) Morphology of MCF10A cells with shCtrl and shp53. Scale bar = 100 μm. (**H**) Immunoblotting of p53 and EMT markers in MCF10A cells with shCtrl and shp53.

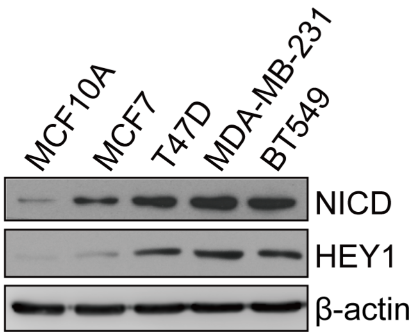

Supplementary Figure S3: Increased Notch activity is observed in TNBC cells. Immunoblotting of NICD and HEY1 in normal MCF10A, luminal (MCF-7 and T47D) and TNBC (MDA-MB-231 and BT549) cell lines.

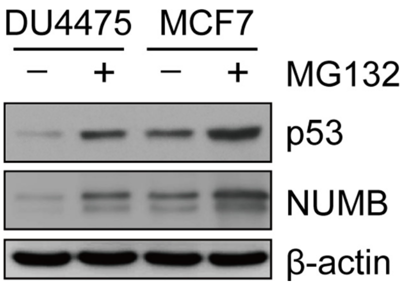

Supplementary Figure S4: Downregulation of NUMB determines decreased p53 in breast cancer expressing wild-type p53. Immunoblotting of NUMB and p53 after treatment with MG132 (10 µM) in DU4475 and MCF7 cells.

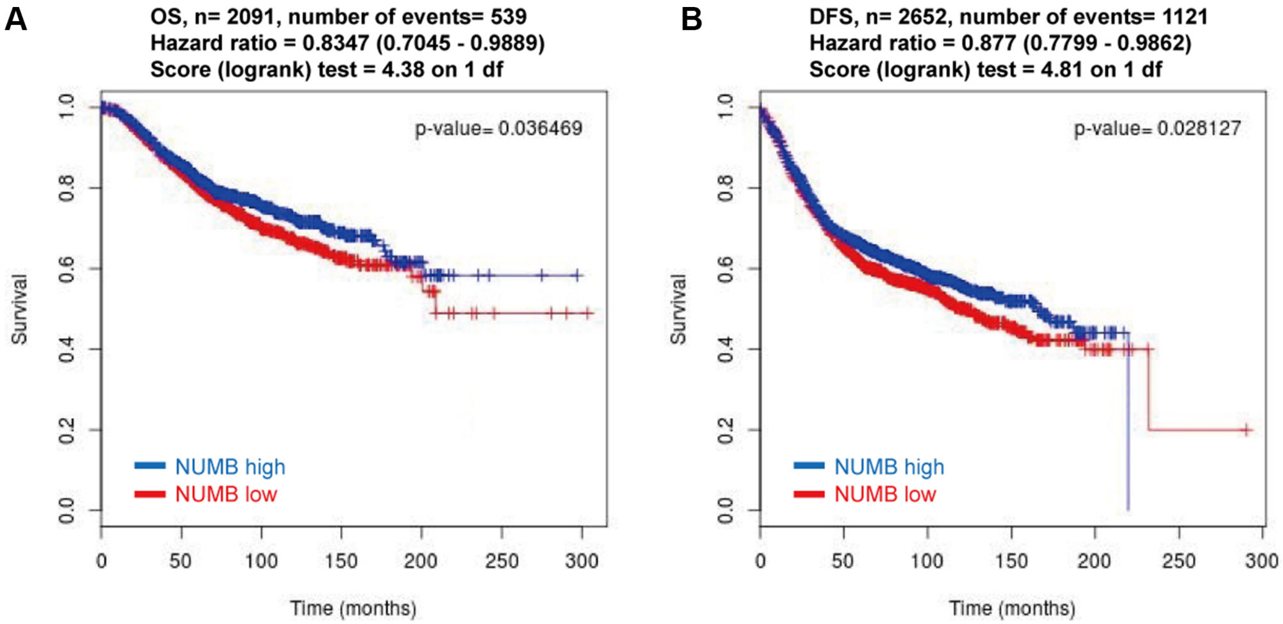

Supplementary Figure S5: High NUMB expression is associated with better survival of breast cancer patients. Kaplan-Meier survival analysis of the publicly datasets for evaluating survival rate of NUMB expression on overall survival (OS) (**A**) and disease-free survival (DFS) (**B**) of breast cancer patients.

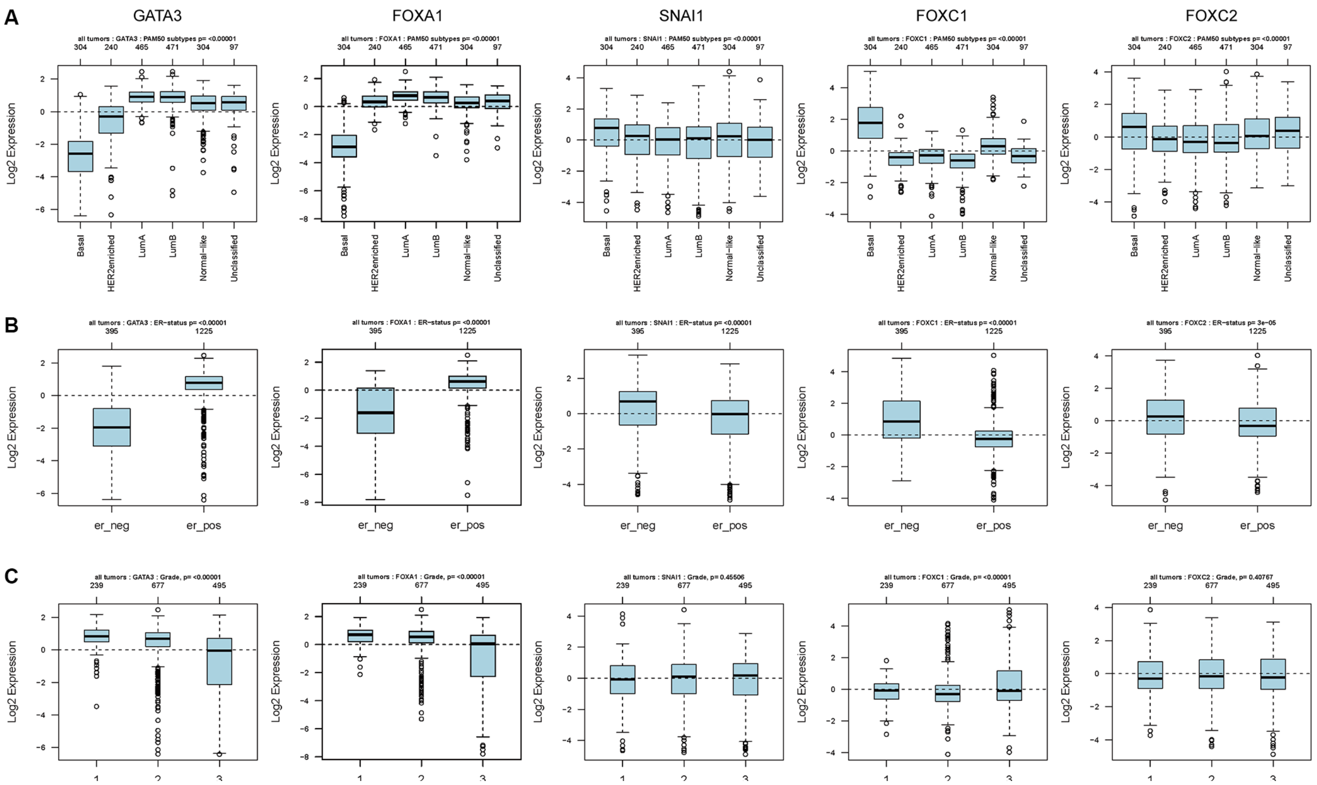

Supplementary Figure S6: The correlation between EMT regulators expression and clinicopathological features of breast cancer. Analysis of the EMT negative regulators (GATA3 and FOXA1) and the EMT inducers (SNAI1, FOXC1 and FOXC2) expression in breast cancer tumors stratified according to PAM50 subtypes (**A**) ER status (**B**) and histologic grade (**C**) using publicly deposited gene expression datasets.
